# Mechanical regulation of the early stages of angiogenesis

**DOI:** 10.1098/rsif.2022.0360

**Published:** 2022-12-07

**Authors:** Sara Barrasa-Ramos, Claire A. Dessalles, Mathieu Hautefeuille, Abdul I. Barakat

**Affiliations:** ^1^ LadHyX, CNRS, Ecole Polytechnique, Institut Polytechnique de Paris, Palaiseau, France; ^2^ Laboratoire de Biologie du Développement (UMR7622), Institut de Biologie Paris Seine, Sorbonne Université, Paris, France; ^3^ Facultad de Ciencias, Universidad Nacional Autónoma de México, CDMX, Mexico

**Keywords:** sprouting angiogenesis, endothelial mechanobiology, shear stress, transmural flow, cell–matrix interaction

## Abstract

Favouring or thwarting the development of a vascular network is essential in fields as diverse as oncology, cardiovascular disease or tissue engineering. As a result, understanding and controlling angiogenesis has become a major scientific challenge. Mechanical factors play a fundamental role in angiogenesis and can potentially be exploited for optimizing the architecture of the resulting vascular network. Largely focusing on *in vitro* systems but also supported by some *in vivo* evidence, the aim of this Highlight Review is dual. First, we describe the current knowledge with particular focus on the effects of fluid and solid mechanical stimuli on the early stages of the angiogenic process, most notably the destabilization of existing vessels and the initiation and elongation of new vessels. Second, we explore inherent difficulties in the field and propose future perspectives on the use of *in vitro* and physics-based modelling to overcome these difficulties.

## 1. Introduction

The vascular system is a multi-scale network of blood vessels perfusing every organ of the body to ensure tissue oxygenation, nutrient delivery and waste product removal. The vasculature is generated through the processes of vasculogenesis and angiogenesis. Vasculogenesis denotes de novo vessel formation by precursor cells or endothelial cells (ECs) distributed within the tissue matrix [1], whereas angiogenesis refers to the emergence of new microvessels from pre-existing vessels [2]. Angiogenesis is the principal mechanism for developmental, regenerative and pathological vessel formation in late embryonic and postnatal stages. It is also essential in large-scale tissue engineering, enabling the transport of oxygen and nutrients beyond their diffusive limits [3]. As such, angiogenesis is an active area of research in a wide range of fields, from fundamental understanding of pathological development to the opening of new avenues in engineered tissue vascularization.

The angiogenic process has often been examined from the perspective of biology and biochemistry, with emphasis on gene expression, metabolism, signalling pathways and the role of different types of cells such as mural and stem cells [4–9]. However, in recent years, the need for embedded vascular networks in tissue engineering has elicited interest in the pursuit of alternative strategies of angiogenic control. The availability of *in vitro* systems and computational models has more recently enabled appreciation of the importance of mechanobiology, with mechanosensing and mechanotransduction as key players [10–13].

The vascular microenvironment is highly dynamic, subjecting ECs to mechanical forces to which they are highly responsive [11,14,15]. Indeed, from intracellular cytoskeletal remodelling to changes in collective behaviour, EC mechanotransduction events intricately regulate numerous aspects of vascular processes including angiogenesis [16–19]. Major biophysical cues for angiogenesis can be broadly classified as either fluid or solid mechanical, emanating from the vessel lumen or the surrounding parenchyma. While cyclic longitudinal and circumferential strain, fluid dynamic shear stress and pressure result from pulsatile blood flow, ECs are additionally continuously subjected to transmural and interstitial flows, and they interact physically with their basement membrane and with adjacent cells.

Here, based principally on *in vitro* evidence, we will highlight how ECs are particularly responsive to mechanical stimulation. We begin by reviewing the role of mechanical forces in the early stages of sprouting angiogenesis, namely vessel destabilization, sprout initiation and elongation. We then focus on the effects of different fluid and solid mechanical stimuli on ECs and discuss their involvement in each stage of angiogenesis. We conclude by addressing some of the challenges and future perspectives in the field, underscoring the coupled nature of these stimuli and their multi-scale character.

## 2. Early phases of sprouting angiogenesis

Sprouting angiogenesis is the formation of new blood vessels or neovessels from a pre-existing vascular network. Angiogenic sprouting is a very important event, not only in the development of organs and tissues, but also in pathophysiological processes involved in tissue repair, wound healing, regeneration, fibrosis and cancer [20]. Sprouting begins with the degradation of the basement membrane and the activation of ECs that ‘sprout’ out of the original vessel and elongate into the extracellular matrix (ECM). At a later stage, the sprout connects with another vessel in a process known as anastomosis [21,22]. The lumen of the sprout is formed in parallel with elongation and anastomosis [23,24]. Ultimately, the vascular network architecture is optimized by vessel pruning [23,25,26]. Although the exact role of mural cells in the angiogenic process is yet to be fully elucidated, we know that together with the deposition of a new basement membrane by ECs, the recruitment of mural cells is necessary for the stability of the nascent angiogenic vessel [27–29].

**Figure 1.**
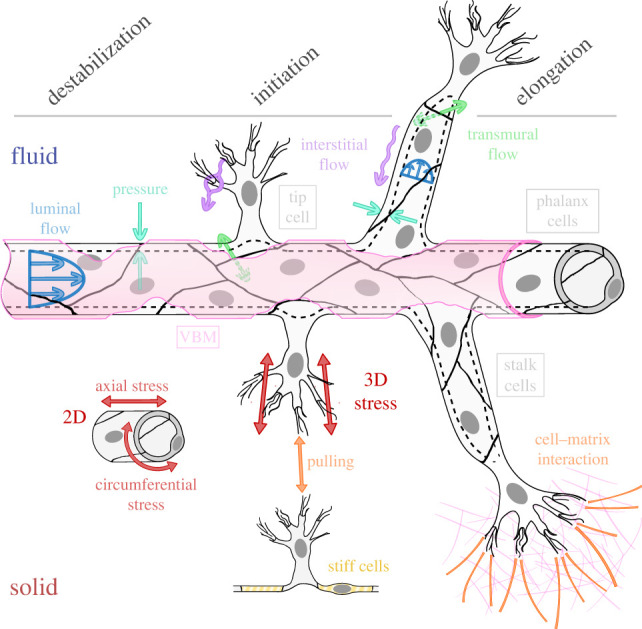
Fluid mechanical (top half) and solid mechanical (bottom half) stimuli during the early stages of angiogenic sprouting. From left to right: destabilization, initiation and elongation. Cold colours represent fluid mechanical stimuli: liminal (blue), transmural (green) and interstitial (purple) flows and pressure; and warm colours, solid mechanical aspects: two-dimensional and three-dimensional stresses (maroon), cell–cell and cell–matrix interaction (orange) and cell stiffness (yellow).

The early events of angiogenesis can be subdivided into three phases: (i) destabilization of the vessel wall, (ii) initiation of the sprout, and (iii) elongation of the sprout. These phenomena are generally consecutive, although initiation may overlap with destabilization. In this section, we describe each of these stages, emphasizing the associated changes in the mechanical environment of ECs (figure 1).

### 2.1. Destabilization of the wall of the original vessel

Destabilization refers to the modifications of the vessel wall's baseline structure that compromise its integrity and enable the onset of angiogenesis. Microvessels are composed of the EC monolayer lining the lumen, the vascular basement membrane (VBM) ensheathing the ECs and sparse mural cells (pericytes or smooth muscle cells) surrounding the vessel. Although the cooperation of these three constituents is essential for vessel stability, we focus here on the destabilization of the VBM and the endothelial lining. The role of mural cells was the subject of a separate recent review [30].

The VBM, a thin specialized ECM [31] on which the endothelium resides [32,33], enables cell anchoring, provides mechanical strength and regulates the transport of growth factors by acting as a reservoir of matrix-bound molecules [29,34]. Destabilization of the VBM occurs through VBM degradation, which is triggered by ECs through the secretion of matrix metalloproteinases (MMPs) that cleave VBM constituent proteins [35].

Destabilization of the endothelial lining is the second facet of this first phase. Cell–cell junctions and cell–matrix adhesions, which drive the cohesive nature of the endothelium and control the establishment of the vascular barrier [36,37], are key in this phase. The importance of cell–cell junctions in angiogenesis is supported by the fact that vascular endothelial growth factor (VEGF), a major pro-angiogenic molecule, is known to disrupt these junctions [38–44]. Monolayer fluidization, defined as an increase in cell motility inside the monolayer that is favoured by weaker intercellular junctions [45], was recently highlighted as an early event in angiogenic sprouting [46]. Moreover, altered cell–cell junctions reduce tissue tension [47] and lead to differential activation of focal adhesions [48], which appears to favour cell motility towards the parenchyma [49]. Finally, alterations in cell–cell junctions and cell–matrix adhesions are intricately involved in both proliferation and migration, essential processes of the subsequent phases of sprouting.

At this stage, a question arises as to the choice of markers or indicators of endothelial lining destabilization. There is no single definitive answer to this question, but we consider increased monolayer permeability as a useful indicator and thus use it as such in this review. This choice is motivated by the following two observations: firstly, and in connection with the previous paragraph, the cell–cell junctions that are altered during vessel destabilization regulate paracellular transport and hence wall permeability; secondly, mechanical stimuli influencing angiogenesis in the vasculature (e.g. wall shear stress) also have a major impact on the permeability of EC monolayers.

From a mechanical perspective, the destabilization of the vascular wall might be expected to lead to increased transmural flow (TF) and vessel compliance. Furthermore, the loss of the VBM exposes the EC monolayer directly to the underlying matrix, which has a lower protein density and thus different mechanical properties [34]. In the light of evidence that substrate stiffness regulates the structure and function of many cell types, including ECs [50–53], these alterations in mechanical properties may play a critical role in the progression of the angiogenic process.

### 2.2. Sprout initiation

After vessel wall destabilization, a sprout is initiated by the invasion of the ECM by one or more ‘tip cells’ [54] that are polarized towards the parenchyma. ECs with a ‘tip cell’ phenotype exhibit low proliferation rates, increased migration [55,56] and increased expression of MMPs [35,57]. The Notch signalling pathway, involved in spatial patterning and lateral inhibition during morphological events, has been considered as key in sprout initiation: its activation inhibits the tip cell phenotype in adjacent cells [58,59], thereby controlling the topology of the network [58,59]. Other signalling pathways that appear to be centrally involved include vascular endothelial growth factor (VEGF) and its receptors as well as the Tie receptors and their ligands, the angiopoeitins [60–62]. An interesting question that remains a matter of debate is the role of mechanics in determining the location within a blood vessel where tip cells form. While the direction of interstitial and TF appears to be critical in determining the circumferential position at which sprouting occurs, luminal shear and pressure appear to be more pertinent for determining the axial position of sprout initiation [63]. Upon loss of the VBM, the tip cells that had previously been adherent to it become immersed within the underlying ECM where they can potentially come in direct physical contact with parenchymal cells. During this process, tip cells shift from a two-dimensional to a three-dimensional environment and are subjected to matrices with different mechanical properties [64] that are in turn modified by the action of these cells [65]. The means by which tip cells probe this new environment for biochemical and mechanical cues constitute an active field of research [66]. It is generally accepted that they do so through actin-rich filopodia [55,57,67,68], although lamellipodia or blunt pseudopodia have been shown to adopt this role in the absence of filopodia [69,70].

### 2.3. Sprout elongation

Once the sprout is initiated, it penetrates the parenchyma, resulting in a cord-like structure, which evolves into a closed-ended tube [71,72]. Tip cells lead the way and induce a ‘stalk cell’ phenotype in adjacent ECs to recruit them as followers in the sprout [55], as opposed to the ‘phalanx cells’, which stay in the original vessel. Interestingly, cell phenotype can change during elongation with tip and stalk cells often switching roles [73–76]. While tip cells spearheading the sprout exhibit an enhanced migratory phenotype with numerous filopodia [58], stalk cells rely on an increased proliferation rate to guarantee the continuity of the network [77,78]. Indeed, angiogenic ECs transition from the phalanx phenotype, one of the most quiescent cells in the body with lifespans of hundreds of days [79], to the stalk phenotype with turnover times on the order of tens of hours [80]. Normal sprout development requires a precise balance between migration and proliferation. An imbalance between these two processes may lead to detached tip cells [81] or to tortuous vessels [82]. The formation of a lumen is concomitant with sprout elongation, with stalk cells of lumenized sprouts expressing luminal–abluminal polarity [83–85]. Although specialized junction-based mechanisms contribute to monolayer integrity [86,87], sprouts have been found to be leaky during elongation [88]. The ensuing increase in TF promotes lumen formation and elicits a small luminal flow (LF) when a lumen already exists [89,90], generating luminal shear stress (LSS) on the cells. Complex flow fields develop within newly formed lumens with plasma recirculation and pressure oscillations due to circulating cells entering the sprout [91,92].

Based on the above, we can define key markers for each sprouting angiogenesis phase that will be used in the rest of the review as readouts for the effect of each of the mechanical forces of interest. More specifically, we use MMP activity and endothelial destabilization as readouts for the destabilization of the original vessel, tip cell induction and Notch signalling as indicators of sprout initiation and EC proliferation, migration and polarization as well as lumen formation as markers of sprout elongation.

## 3. Role of fluid mechanics in angiogenic sprouting

As in atherosclerotic lesion development and aneurysm formation [93], flow-derived forces have been shown to play a key role in angiogenesis [11]. In a quiescent vascular network, the combination of luminal, transmural and interstitial flow (IF) paths ensures the transport of oxygen, nutrients and waste products beyond diffusive distances [3]. Changes in these flow paths induced by either developmental or pathological processes can trigger angiogenic responses that extensively remodel the vascular architecture and hence in turn alter the flow and transport environment [94–96]. In what follows, we present flow-derived cues and discuss the role that they have been reported to play in angiogenic sprouting. This information is summarized in table 1.

**Table 1.** Summary of the effects of fluid mechanical stimuli on the early stages of angiogenesis. Effects are listed as positive for angiogenesis when increased. The first column shows the reviewed mechanical cues: LF, luminal flow; TF, transmural flow; IF, interstitial flow; P, pressure; PF, pulsatile flow. The second column further subdivides the stimuli to improve readability: AB, apical-to-basal; BA, basal-to-apical; Up/Contra, upstream of the original vessel/contradirectional to the sprout; Down/Co, downstream of the original vessel/codirectional to the sprout; HP, hydrostatic pressure; IP, interstitial pressure; τ shear; P, pressure. Colour code: presence (black), low (blue)/physiological (brown)/high (purple), but for pulsatile flow where non-reversing (pink)/reversing (green)/oscillatory (dark blue). Symbols: ? no information, ↑ increase, ↓ decrease, × contradictory effects, - no effect/minor effect, ⊲ gradient, → preferred direction. Striped background means hypothesis. Growing arrows imply magnitude correlation. In each cell, values of the stimulus increase from top to bottom.

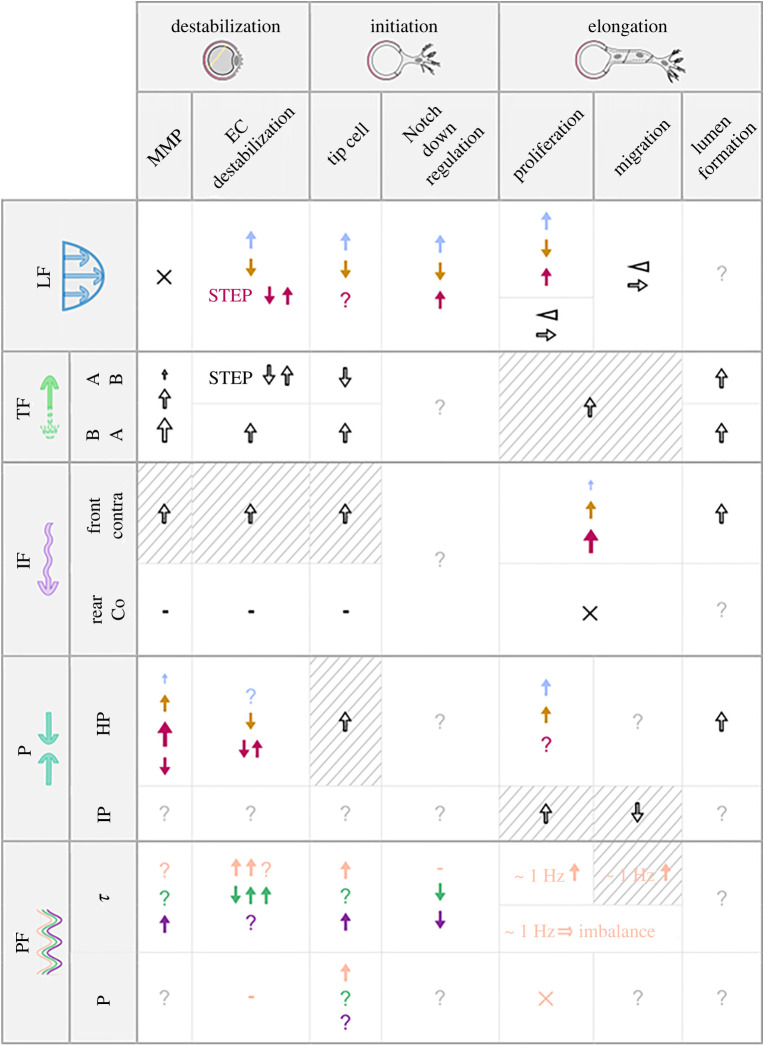

### 3.1. Luminal flow

The LF of viscous blood generates a tangential shear (frictional) stress on the EC apical surface (wall shear stress; WSS). Although large variations have been reported [97,98], physiological ranges of WSS in human circulation are 1–6 dyn cm^−2^ on the venous side and 10–70 dyn cm^−2^ on the arterial side, with the highest shear stress levels in the microvasculature [99,100] (note that in line with convention in this field, shear stress values in this manuscript are expressed in dyn cm^−2^; recall that 1 dyn cm^−2^ = 0.1 Pa). WSS in a vessel depends on blood viscosity, flow rate and vessel radius, all of which are affected by diseases that are associated with altered angiogenesis such as obesity and hypertension [101,102]. Moreover, circulating cells [103,104], curved segments [105], bifurcations, merging branches [106,107] and pulsatility all affect local WSS values.

WSS effects on angiogenesis are controversial. *In vivo,* sprouting has been correlated with both low [108–110] and high levels of WSS [111,112]. *In vitro,* WSS levels ranging from 3 to 15 dyn cm^−2^ have been reported to both attenuate [67,113] and enhance sprouting [114,115]. Moreover, the effect of WSS appears to be different for arteries and veins, with only venous EC angiogenesis and tubulogenesis being inhibited by increased WSS [108,109].

*In vivo,* vessel stability is compromised by changes in WSS [93]. *In vitro,* the effects of WSS on VBM degradation are unclear, although endothelial stability appears to be favoured by physiological levels of steady WSS (8–15 dyn cm^−2^) [116]. Interestingly, the effects of WSS appear to strongly depend on the biochemical environment, as exemplified by the strong synergistic effect with sphingosine 1-phosphate (S1P) [114,117,118]. In the specific case of basement membrane degradation, MMP levels have been reported to either increase [115,119,120] or decrease [121–123] with increased WSS. A particularly interesting finding is the possible presence of a maximum in MMP activation and matrix invasion, and thus VBM degradation rate, at physiological WSS levels (5.3 dyn cm^−2^) [117]. Regarding the integrity of the endothelium, the effects of WSS depend on the duration, rate and magnitude of the shear stress as well as on the vascular bed [124]. In arterial and microvascular ECs, an increase in WSS, within the physiological range, leads to increased permeability in the first 1 to 3 h [125–128] but appears to enhance monolayer integrity after that [129–131]. By contrast, both in the short and the long term, low values of WSS induce disorganized junctions and increased permeability [129,132]. In venous ECs, while low-end physiological levels of WSS (less than 10 dyn cm^−2^) improve barrier function [133], an acute increase in WSS induces a transient increase in permeability, with discontinuous cell–cell junctions, for WSS values ranging from 4 to 20 dyn cm^−2^ [134,135]. *In vivo*, similar differences have been reported between low- and high-flow vessels [136–138], although the results remain somewhat controversial [139]. These opposite short- and long-term effects on vessel destabilization may explain the contradictory results of WSS on sprouting found *in vitro*.

The initiation of a sprout through tip cell selection is favoured by sub-physiological WSS levels, whereas physiological shear levels induce a more quiescent behaviour. Recently, it was shown that no or low WSS promotes the formation of new vascular branching points [121,140]. This is in line with the proposed paradigm that loss of shear stress modifies gene expression, transforming phalanx cells into tip cells [141]. Notch signalling, whose activation favours the phalanx and stalk cell phenotypes [58,59] and limits branch formation [142–144], is activated at physiological WSS levels [108,145], especially in venous ECs [146], although downregulation has been observed for WSS values above 10 dyn cm^−2^ [147].

Because the distal tips of vascular sprouts are closed-ended, it is tempting to posit that sprout elongation is independent of luminal WSS. However, several studies show increased sprout lengths in networks exposed to shear stress [115,148,149]. Compared with static (no flow) conditions, very low WSS levels of 10^−4^ to 10^−3^ dyn cm^−2^ have been shown to favour EC proliferation [92], whereas physiological values of 15–20 dyn cm^−2^ inhibit glycolysis and DNA synthesis, which are necessary for cell proliferation [150–153]. Higher values up to 100 dyn cm^−2^ increase proliferation and decrease apoptosis in a magnitude-dependent manner [154], although the apoptosis trend gets reversed, increasing above 300 dyn cm^−2^ [155].

**Figure 2.**
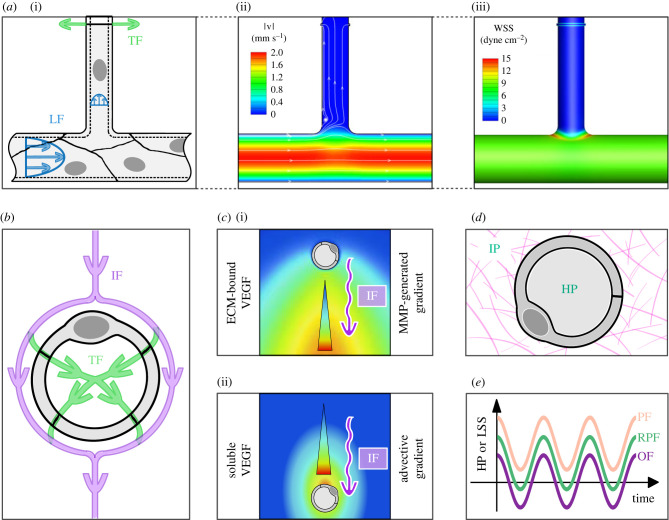
Specificities of some fluid mechanical stimuli. (*a*) Maximum shear stress is found at the base of sprouts when TF is considered: (i) schematic of luminal (LF) and transmural flows (TF), (ii, iii) flow velocity and resulting wall shear stress (WSS) fields (adapted from [90]). (*b*) Parallelism between the effects of IF around the entire microvessel and those of TF around a single EC. (*c*) IF generates opposite gradients of (i) matrix-bound (adapted from [156]—Copyright (2005) National Academy of Sciences, USA) and (ii) soluble (qualitative) VEGF isoforms. (*d*) Localization of hydrostatic pressure (HP) and interstitial pressure (IP). (*e*) Example waveforms of non-reversing pulsatile flow (NPF), reversing pulsatile flow (RPF) and oscillatory flow (OF).

Numerical simulations indicate that WSS maxima can be found at the base of sprouts [90] (figure 2*a*). When combined with the observation of increased EC migration from low to high shear stress regions [138], this finding points to a reinforcement of the migration of ECs in the original vessel towards the base of the new sprout. Although this is potentially suggestive of an elongation mechanism, whether or not these migrating cells end up feeding the sprout would depend on other cues taking the lead within the sprout itself, where the WSS is very low. Another important consideration is that temporal and spatial shear gradients induce different EC proliferative behaviours, depending on the mean WSS level and the sign of the gradient. Positive temporal gradients (as occurs in flow onset) have been shown to stimulate EC proliferation [157]. Spatial gradients appear to have different effects depending on mean shear stress level. The threshold value of WSS is probably dependent on EC type (arterial or venous) but falls in the range of 10–30 dyn cm^−2^. Below this threshold, there is contrasting evidence on the effects of spatial gradients, with some studies reporting no significant effect [157] and others showing altered cell–cell junctions [158]. Above this threshold, EC proliferation has been reported to be favoured by positive spatial WSS gradients and hindered by negative gradients [155]. We therefore hypothesize that temporal and spatial WSS gradients are critical determinants of sprout elongation.

### 3.2. Transmural flow

TF is generated by the intra- or extravasation of blood plasma that arises from the pressure difference across the vessel wall. In capillaries, hydrostatic and osmotic transmural pressure differences add up to 0.5–3 mm Hg [159,160], thereby inducing fluid extravasation. The equivalent values in arterioles and venules are around 15 and −15 mm Hg, respectively [161,162]. Estimations of the resulting shear stress on cell–cell junctions vary from 0.1 dyn cm^−2^ [63] to 50 dyn cm^−2^ [163,164]. Several factors that are critical for transmural fluid flow including vessel permeability, interstitial fluid pressure and blood pressure are altered by pathologies such as cancer [160,165], hypertension [166] and stroke [167]. By virtue of its flow across the vascular wall, TF is likely to coax ECs to abandon their monolayer state and migrate into the underlying tissue.

*In vivo* experiments suggest that sprouts originate from microvessels under the influence of inward TF [63], consistent with the fact that tumour angiogenesis occurs preferentially on the venous side of the circulation. Indeed, there is fairly broad consensus that at the EC level, basal-to-apical (inward) TF increases sprouting [67,168,169]. Conversely, the effect of apical-to-basal (outward) TF is more controversial. While some studies have reported a positive effect on sprouting [106,115,170], others suggest that outward TF stabilizes vessels [129] and inhibits sprouting [168].

Although the mechanisms underlying TF-mediated vessel destabilization remain incompletely understood, a synergistic effect with biochemical pro-angiogenic factors appears to play a role [156,170–172]. For instance, some studies have reported upregulation of MMP expression by TF [173] in a magnitude-dependent fashion [115], leading to proportionate degradation of the ECM [169]. The effect of TF on the integrity of the endothelium appears to be more complex and to depend on flow direction. More specifically, in the short term (2 h), a sudden increase in apical-to-basal pressure difference leads to a significant reduction in endothelial permeability [174]. However, in the longer term, the resulting shear stress on cell–cell junctions leads to an increase in permeability [163]. By contrast, basal-to-apical TF disrupts cell–cell junctions and reinforces cell–matrix adhesion [168,170], thereby destabilizing the monolayer [129].

While the VBM does not seem to be a key player in fluid transport [175], EC monolayer destabilization may increase TF during the sprout initiation phase. ECs subjected to basal-to-apical TF exhibit polarization in the form of actin accumulation and protrusion formation, characteristic of the tip cell phenotype, on the upstream (i.e. abluminal) side [168]. The underlying mechanism might be similar to drag-induced migration experienced by cancer cells in three-dimensional substrates [48], with an additional effect due to cell-matrix interactions of two-dimensional monolayers being limited to the abluminal side. Indeed, sprouts under apical-to-basal flow exhibit fewer filopodia [67,170]. Although LF has been shown to modulate Notch activity, the effects of TF on the Notch pathway have, to the best of our knowledge, not been studied. An interesting question is whether or not TF provides a possible link between hypoxia-induced hyperpermeability [176] and hypoxia-induced Notch signalling [177].

TF is strongly coupled to IF, hampering efforts aimed at separating their respective effects on sprout elongation [88,169,170]. However, in lumenized sprouts, TF allows sprout luminal perfusion [90] (figure 2*a*), inhibiting sprout regression until anastomosis occurs [88,115,138]. As such, TF determines the flow pattern within the sprout and the ensuing luminal shear stress [90]. Interestingly, it has been suggested that due to the very narrow junctional spacing, the shear stress exerted on cell–cell junctions as a result of TF is comparable to that resulting from luminal WSS [163]. Inward flow reinforces EC apicobasal polarity, which is key to angiogenic sprouting [168,178] and apical-to-basal pressure drop increases the size of the newly formed sprout lumen [169].

### 3.3. Interstitial flow

IF is the movement of fluid within the parenchyma due to pressure differences between the ECM, on the one hand, and the blood and lymph circulatory systems, on the other [179]. *In vivo* quantification of IF is limited, with reported velocities of up to 2.0 µm s^−1^ [180,181]. Flow through the porous ECM results in shear and pressure forces on the abluminal surfaces of both the original and newly formed vessel walls. Mean pressure drops across a microvessel as a result of the circumferential IF (figure 2*b*) are estimated to be of the order of 10–100 dyn cm^−2^ [48] with associated shear stresses of 0.001–0.1 dyn cm^−2^, estimated using either a homogenized/mesoscopic model [48,63,169] or a microscopic model of the fibrous ECM [156,182]. Recent results, however, suggest that these levels evolve in both time and space with considerable levels of uncertainty [183]. Conditions that modify IF, such as cardiovascular disease, neoplasia and inflammation, are known to affect vascular development [184–186].

Angiogenic activity is greatly stimulated by IF in the presence of growth factors or other cells but not otherwise [171,187,188]. This coupling is corroborated by the fact that vasculogenesis appears to only be initiated for values of the Peclet number larger than 10 (i.e. with convective transport dominating diffusion), indicating the importance of mass transport considerations [133,189]. IF suppresses co-directional sprouts and enhances contra-directional sprouts both *in vivo* [63] and *in vitro* [171,188]. The IF component perpendicular to the vessel axis [181] might be responsible for this orientation bias (figure 2*c*): similarly to what happens to isolated cells under IF [48], the ‘front’ of the vessel is subjected to an impinging flow with associated high pressure, while the ‘rear’ sees a lower pressure due to losses through the ECM (figure 2*b*). This asymmetry may conceivably lead to differential activation of ECs and directionally biased angiogenesis. Additionally, even at Peclet numbers as small as 0.5, mass transport of proteases and VEGF distribution are both strongly influenced by advective IF [156]. The spatial pattern of VEGF, both soluble and matrix-bound, is known to influence angiogenesis [190]. Therefore, IF may also regulate angiogenesis indirectly through its effect on VEGF distribution [156,168,188].

Although definitive evidence remains elusive, we propose that the upstream accumulation of VEGF and MMP by the impinging IF [168] constitutes a potential mechanism by which IF regulates vessel destabilization. High concentrations of VEGF would induce wall destabilization by disrupting cell-matrix and cell–cell junctions [43,191], and MMP accumulation would enhance ECM degradation [192]. Additionally, MMP secretion and activation might also be increased by IF-induced shear in ECs, as has been demonstrated for both smooth muscle cells and fibroblasts [193,194].

Sprout initiation through induction of the tip cell phenotype appears to be directional. Sprouts have been reported to grow against the IF direction, and sprouting is lost after IF removal or reversal [170,171]. The role of VEGF in tip cell selection and Notch regulation [195] points to mass transport as a major player during this stage. Surprisingly, however, IF-generated VEGF distributions do not appear to be consistent with the counter-IF direction of sprouting. In the case of matrix-bound VEGF, cleavage by soluble MMPs released by the ECs would be expected to engender lower VEGF concentrations near the blood vessel, and a positive downstream VEGF gradient [190,196] (figure 2*c*(i)). This gradient would, in turn, be expected to favour co-IF sprouting. However, the cleaving of VEGF by single cells appears to be minimal [197], suggesting this autocrine mechanism may play only a secondary role at the capillary level. In the case of soluble VEGF, the upstream impinging IF leads to accumulation at and around the stagnation point [168] (figure 2*c*(ii)); therefore, counter-IF sprouting would imply sprouting towards lower VEGF concentrations, and would be unexpected. In summary, the predicted VEGF gradients would be expected to favour sprouting in the downstream rather than the upstream IF direction. The explanation to the appearance of counter-IF sprouting may thus lie in its induction by mechanical stimuli resulting from IF, with impingement (stagnation) points exhibiting singular behaviours such as pressure maxima or zeros in shear stress and its gradient.

During the elongation phase, IF correlates with the sprout penetration rate and its direction; it also plays a central role in determining sprout morphology. Sprout elongation rate has been shown in several studies to correlate with IF magnitude [63,169,170], possibly through MMP activation and regulation of tip cell migration [169,170], while one study points to a magnitude-independent role of IF [188]. Moreover, IF guides elongation, whether co- [67,106,115] or contra-directionally [63,67,169,171,188], even dominating the effect of VEGF gradients [63]. However, removal or inversion of contra-directional IF results in sprout regression, perhaps linked to a loss of polarization in the stalk cells [138] but mostly to tip cell depolarization as illustrated by filopodial loss [170,171]. Finally, a fine balance between VEGF spatial distribution and IF magnitude is necessary for the formation of continuous and lumenized sprouts [156,169,170,198], controlling their length and thickness [55,171]. Shallow gradients of VEGF improve proliferation and decrease migration, whereas steep gradients have the opposite effect, favouring branching over elongation [199]. Indeed, mass transport resulting from a single IF profile can lead to more or less steep gradients of different VEGF isoforms, depending on their molecular weight and matrix affinity [190], potentially promoting proliferation of the stalk cells and migration of the tip cells at the same time. The distribution of different VEGF isoforms under diffusive conditions has been addressed using computational modelling [200], shedding light on its role in the angiogenic process. Incorporating advection into these studies promises to provide additional valuable insight.

### 3.4. Pressure

ECs are subjected to two types of pressure: hydraulic pressure (HP) from the luminal blood flow on their apical surface and interstitial pressure (IP) from the tissue fluid flow on their basal surface (figure 2*d*). Values of HP in microvessels range from 12 mm Hg in venules to 45 mm Hg in arterioles under physiological conditions [161] and increase in diseases such as diabetes or essential hypertension [166,201] with median values increasing by up to 20% in skin capillaries [202]. Physiological values of IP are in the range of −8 to 6 mm Hg depending on the organ but can reach values as low as −30 mm Hg in burned tissue and up to 60 mm Hg in the centres of tumours [160,203].

*In vivo*, sprouting appears to preferentially occur from lower-HP vessels towards higher-HP vessels [110]. Furthermore, hypertension has been linked to sprouting impairment [204]. High IP in tumour centres results in avascular zones, although the focus in this regard has been on the expression of biochemical cues by cancer cells rather than mechanical effects on ECs [184]. The literature on pressure and angiogenesis *in vitro* is limited, with only one report of increased sprouting at the low end of the physiological pressure range [205]. Interestingly, in *ex vivo* experiments, veins exhibit sprouting in response to arterial flow [112] but not to arterial pressure alone (i.e. without the increasing shear) [206]. This finding suggests that pressure plays a secondary role to other stimuli, such as luminal shear or TF. Interestingly, sprouting is observed at abluminal impingement sites where IP is high, but it is hampered at vessel bifurcations [106] and merging points [110], which represent local maxima in HP.

Data scarcity precludes outright interpretation; however, microvessel stability appears to be favoured by physiological levels of pressure but compromised under pathologically high values. In bovine aortic ECs *in vitro*, MMP production is enhanced under pressures up to 150 mm Hg although it declines afterwards (well above the characteristic values for microvessels but close to the physiological maximum for bovine aortic systolic pressure [207]), with Piezo-type mechanosensitive ion channel component 1 (Piezo1) probably mediating the entire process [208–210]. Physiological levels of microvascular HP (10 mm Hg) improve monolayer integrity [211], while pathological levels of blood pressure increase vessel permeability in mice through disruption of adherens junctions [209]. Interestingly, the effects of arterial pressure (50–100 mm Hg) on cell–cell junctions appear to be time-dependent: adherens junctions are reinforced during the first hour but weaken afterwards, resulting in intercellular gap formation [209,212–214].

The direct effects of pressure on tip cell selection or Notch signalling have received little attention. Nonetheless, sprout initiation hinges on changes in both the tip cell cytoskeleton to form protrusions and cell–matrix junctions to initiate migration. Arguably, pressure participates in the regulation of both of these processes. More specifically, exposure of bovine aortic and pulmonary ECs to pressure steps ranging from 10 to 150 mm Hg leads to cytoskeletal remodelling, with recruitment of thick actin fibres to central regions [213,215,216] and multi-layering of F-actin filaments [217]. Pathological levels of hydrostatic pressure also increase the tortuosity of the contours of individual ECs [216], which might be linked to protrusion formation. The effects on cell–matrix adhesion are more controversial: while physiological values (3 mm Hg) reinforce focal adhesions [125,126], no changes in focal adhesion dynamics have been observed under pathological conditions (100 mm Hg) [212].

Sprout elongation relies principally on stalk cell proliferation, which has been shown to be modulated by pressure, and on tip cell migration for which the effects of pressure have yet to be explored. Physiological levels of HP induce proliferation of venous ECs [125,126,214] through reduced cellular contact inhibition resulting from the disruption of adherens junctions [212,213]. In arterial ECs, proliferation is enhanced for low values of HP [205,215], but the repercussions of higher pressures are unclear [205,218]. Although the effects of pressure on tip cell three-dimensional migration have not yet been studied, we propose that pressure values that stimulate EC proliferation but not tip cell migration might be in part responsible for the increased tortuosity in tumour-feeding vessels [219,220] through a buckling-like mechanism. Additionally, lumens can develop through inverse membrane blebbing [54], a blood pressure-induced mechanism, and are probably enlarged as MMP activity is enhanced [208,221]. Recent evidence points to a fundamental role for pressure in the formation of junctional fingers, protecting from blood leakage during lumen expansion [222]. As for IP, improved vasculogenesis under 50 mm Hg [223] suggests a potential effect on sprout elongation, a topic that certainly merits further investigation.

### 3.5. Flow pulsatility

Blood flow is pulsatile with baseline heart rates typically in the range of 40–100 bpm and exceeding 200 bpm during exercise, which translates to frequencies in the range of 0.7–3.3 Hz. Although pulsatility is partially dampened throughout the arterial vascular tree, several studies suggest the persistence of significant unsteadiness in the microvasculature even down to capillaries in various vascular beds [200,224–228]. Conceptually, pulsatile flow waveforms can be non-reversing, with positive values throughout the entire cardiac cycle; reversing, with a positive mean value but with negative (or reverse) flow during a portion of the cycle; or purely oscillatory, with periodic fluctuations around a zero mean value (figure 2*e*). Flow pulsatility translates into pressure and WSS oscillations.

Although the literature on the effect of flow pulsatility on angiogenesis is sparse, the few studies that exist suggest an effect that differs from that of steady flow. For instance, unlike steady shear, oscillatory shear stimulates tubulogenesis in venous and microvascular ECs but not in arterial ECs [109]. Interestingly, pulsatility appears to be insufficient to induce angiogenesis on its own [206], with pulsatile shear even limiting sprouting compared with steady shear [81]. Fluctuations may also affect mass transport and autocrine signalling [229], with effects on angiogenesis that remain unknown.

The effect of flow unsteadiness on vessel stability remains unclear, but most results point to pulsatile shear stress as a destabilizing factor and to pulsatile pressure having a more limited effect. Little information is available on the role of unsteady flow on MMPs, with one study pointing to upregulation under oscillatory shear [230]. As for EC monolayers, non-reversing pulsatile flow (NPF) has been found to increase monolayer permeability within the first 3 h, contrary to reversing pulsatile flow (RPF) under which this increase was suppressed [231]. Surprisingly, however, both reversing and NPF have been shown to disrupt cell–cell junctions within the first 6 h [232]. For longer exposure times, RPF results in significant disruption, whereas the effects of NPF remain unclear [232–234]. Furthermore, the effects of unsteady shear stress are frequency dependent, with permeability increasing significantly for 1 Hz but not 0.1 Hz oscillatory flow (OF) [235]. Pulsatile pressure appears to have a less pronounced effect on monolayer barrier function than pulsatile shear stress [236].

The effect of pulsatile flow on sprout initiation has seldom been considered, although it is known that pulsatile flow can induce extensive cytoskeletal remodelling and upregulate Notch signalling. On the one hand, alterations in stress fibre and peripheral actin distribution under both reversing and non-reversing pulsatile shear [237,238] as well as under pulsatile pressure [236] suggest a possible role for these stimuli in tip cell phenotype specification. On the other hand, compared with steady flow, RPF and OF induce Notch upregulation while NPF does not [147]. However, the overall picture is clouded by results from a three-dimensional network formation study that reported no significant differences in the number of branches and bifurcation points under pulsatile flows of different magnitudes and frequencies [81].

During the sprout elongation phase, the effects of unsteady shear stress and pressure on the necessary balance between migration and proliferation remain unclear. While 1 Hz non-reversing pulsatile shear appears to maximize EC proliferation [239], it has been shown to disrupt the migration-proliferation equilibrium, leading to detached tip cells [81]. The effects of pressure fluctuations on sprout elongation are equally complex, with pulsatile pressures with amplitudes of 40 mm Hg either promoting or impairing EC proliferation, depending on the mean pressure value [240,241].

## 4. Role of solid mechanics in angiogenic sprouting

In addition to fluid forces/stresses, ECs are continuously subjected to mechanical stimuli from their solid surroundings, to which they respond and adapt. These mechanical interactions are bidirectional, reciprocal and coupled, complicating our understanding of their effects on ECs in general and on the angiogenesis process in particular. Several pathologies are linked to either excessive neovessel formation (cancer, arthritis, loss of sight) or insufficient angiogenesis (hypertension, ischaemia). In many of these pathologies, alterations in the mechanical properties of the vascular microenvironment have been identified and are generally associated with upregulation or inhibition of angiogenesis [242]. *In vivo,* the organization and overall structure of the vascular microenvironment are shaped by adjacent tissues [243]. Starting from development, a simultaneous and coordinated morphogenesis of organs and their vasculature leads to the emergence of the endothelium, which then adjusts locally to form networks that meet the specific needs of the tissues it penetrates and irrigates [244]. Tissue deformation and loading of the surrounding matrix play an important role in defining the architecture of these vascular networks by inducing alterations in vessel formation, growth and vascular remodelling [245]. In this section, we recapitulate the influence of solid mechanical cues on angiogenic sprouting. This information is summarized in table 2.

**Table 2.** Summary of the effects of solid mechanical stimuli on the early stages of angiogenesis. Effects are listed as positive for angiogenesis when increased. The second column further subdivides the stimuli to improve readability: late and early cyclic compression are as defined in [246]. Colour code: presence (black), low (blue)/physiological (brown)/high (purple). Symbols: ? no information, ↑ increase, ↓ decrease, × contradictory effects, - no effect/minor effect, ⊲ gradient, || parallel, ⊥ perpendicular. Striped background means hypothesis. Growing arrows imply magnitude correlation.

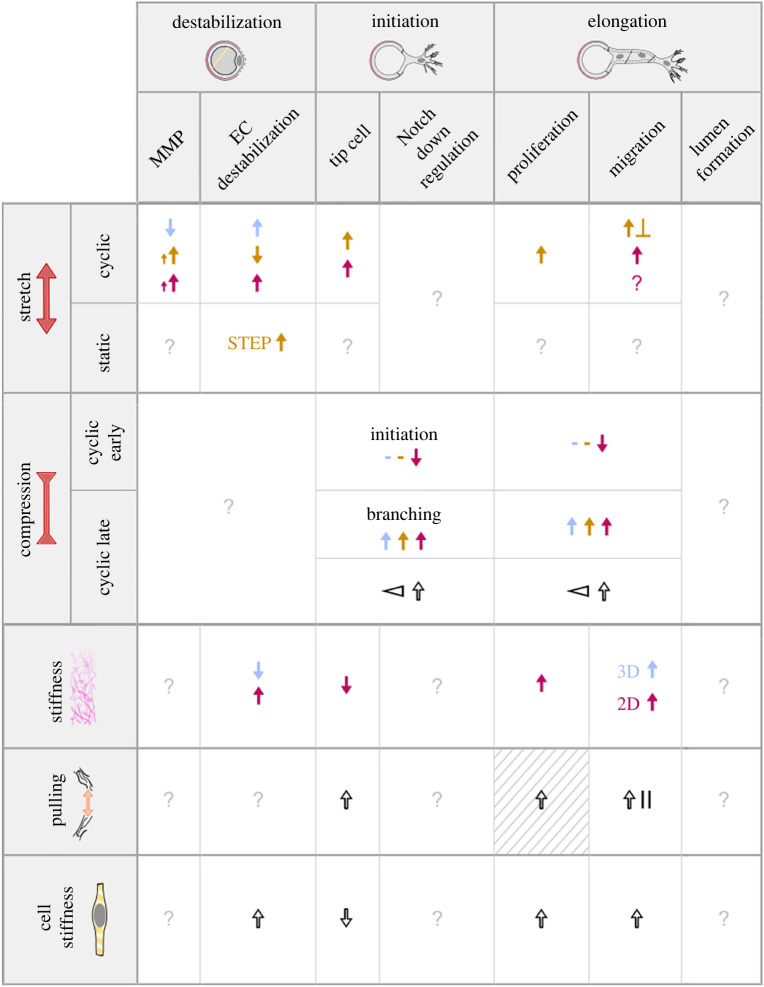

### 4.1. Tensile stress

In the vasculature, ECs are naturally subjected to tensile stresses that can be axial or circumferential, depending on their origin. Axial strains are generated by tissue growth and movement, in particular in the heart, muscles and lungs, while cyclic pulse pressure strains the vessels circumferentially, typically up to 7% [247]. It is generally accepted that tensile stresses are restricted to specific ranges of values to preserve homeostasis. Strains of approximately 5–10% are physiological, whereas strains of approximately 15–20% are considered pathological. Equiaxial chronic cyclic strains as low as 5% at 1 Hz have been found to promote a twofold increase in angiogenesis in ECs *in vitro* [248]. Interestingly, while angiogenesis is a process typically governed by ischaemia of the surrounding tissues [249], one of the main by-products of ischaemia [250], hypoxia-inducible factor 1 (HIF1), is now known to be regulated by mechanical loading through upregulation of mechanosensitive transcription factors [251]. For instance, under non-hypoxic conditions, HIF1 mRNA levels in rats increase after prolonged stretching [252,253].

The activation of ECs by cyclic strain is usually accompanied by MMP activation, critical for blood vessel remodelling [254]. Intermediate levels of strain (10–15%) increase tubulogenesis and endothelial sprouting [255,256], while large strains (above 15%) increase MMP secretion in ECs, suggesting that mechanical forces play a fundamental role in BM degradation events [257]. Interestingly, angiogenesis and MMP expression in ECs under chronic exposure to cyclic strain (1 Hz, 24%) appear to increase with stimulation time [258]. In [259], a Matrigel matrix was used for vessel formation *in vitro* and a negative impact of exogenous cyclic strain was demonstrated in the context of tissue repair and revascularization under this type of mechanical stimulus. Although pro-angiogenic MMP levels were not modified and VEGF signals were even increased under such a stimulus, the final network length ended up being smaller when compared with static conditions. Permeability regulation, a critical EC function, is also impacted by tensile stresses. In a pulmonary endothelium *in vitro* model, permeability was seen to increase in a monolayer stimulated with cyclic strain, caused by the loss of cell integrity due to discrepancies in latero-basal reinforcement of adhesion sites [260]. Interestingly, the amplitude [261] and time dynamics [246] of the mechanical stimuli also appear to be very important phenomenological parameters impacting physiological processes such as permeability and inflammatory signalling by ECs. Compared with static controls, a 1 Hz 20% stretch significantly increased the density of endothelial sprouts [262]. Cyclic strains were also shown to trigger the secretion of several angiogenic factors without affecting VEGF levels [263], suggesting that mechanics is not only as pertinent as biochemical signalling but that it may also cause it. In further support of this notion, the exposure to growth factors, which are responsible for the transition between maintenance and regression of new vessels [264], is now known to be initiated by mechanical deformations and stresses that emanate from the matrix [263].

Tensile stresses also influence cell proliferation and migration. Cyclic circumferential strains of low amplitudes (5–10%) are known to activate ECs, inhibit apoptosis and increase proliferation via cell–cell junctions and signalling [265–268]. By contrast, large strains (15–20%) have the opposite effect [255,269–271], and intermediate levels of strain (10–15%) seem to increase endothelial motility and migration [248]. Generally, tensile stresses align the newly formed sprouts orthogonal to the resulting strain direction [272]. Recent results show that cell–cell junctional tension is also increased during lumen expansion occurring in angiogenesis, and this occurs in a blood pressure-dependent manner. This mechanoresponse causes a force-dependent vinculin recruitment thought to protect cell–cell contacts and to maintain vascular integrity during sprouting [222].

### 4.2. Matrix stiffness properties

**Figure 3.**
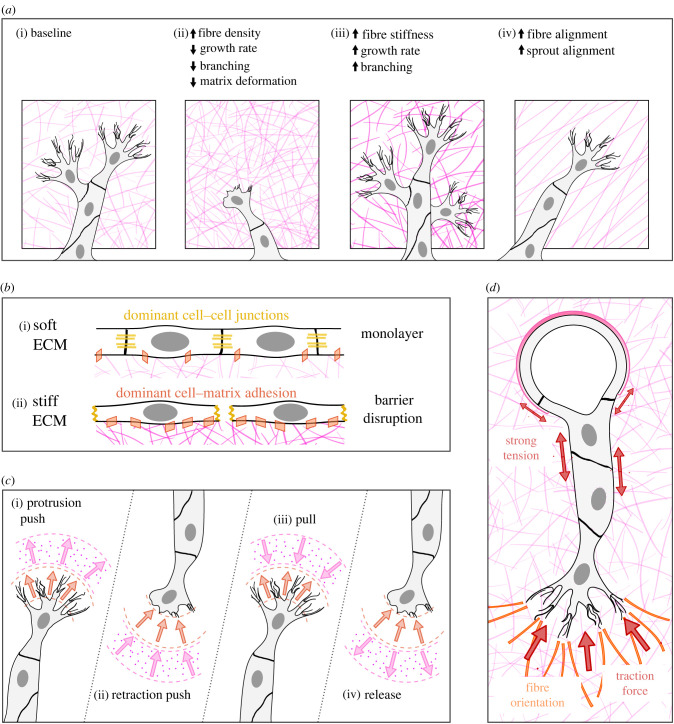
Specificities of some solid mechanical stimuli. (*a*) Effects of increased fibre density, stiffness and alignment on angiogenic sprouting (adapted from [275]). (*b*) Effects of ECM stiffness on cell–cell and cell–matrix junctions. (*c*) Different observed interactions between cell protrusions and the ECM: protrusion displacement in orange and matrix displacement in pink (adapted from [276]). (*d*) Tension in the sprout cell–cell junctions and forces between the tip cell and the extracellular matrix (ECM). The latter induces ECM fibre orientation.

Matrix stiffness is another mechanical stimulus known to affect the formation and structure of angiogenic vessels, both *in vivo* and *in vitro* [273,274]. Recent studies suggest that other mechanical properties of the matrix such as porosity, plasticity or the presence of fibrous constituents, are also probably crucial (figure 3*a*). In a healthy endothelium, the surrounding ECM is principally composed of laminin, type IV collagen and proteoglycans, with low fibronectin levels. In damaged tissues, however, accumulation of fibronectin is observed [277].

As for other cellular and tissue-level processes involved in pathophysiological morphogenesis, stiffness is typically considered one of the principal arbiters, and its influence can be studied thanks to recent progress in the fields of biomaterials and mechanobiology. Studies using hydrogels of varying stiffnesses within the physiological range, from 100 Pa to a few kPa, show a general trend of enhanced vascular network formation in softer matrices. In [278], three-dimensional-encapsulated ECs were shown to spread more and to form longer-lasting vascular networks inside softer MMP-degradable RGDS adhesive peptide hydrogels, while stiffer gels slowed this process greatly. This effect is not gel-dependent as other biomaterials exhibited similar results [279–281]. In [280], it was shown that the EC response to stiffness is cell-specific, as is the final vessel network architecture. This is explained by differences in cell contractility and ECM deformability. Similarly, the mechanical properties of the matrix were found to influence the formation of de novo functional endothelial tubes in a vasculogenesis assay on soft compliant substrates. In [282], the existence of a compliant range of stiffness on which ECs can better self-assemble into network-like structures was identified, related to the appropriate level of cell traction force required for the balance between migration and proliferation. Together with these intrinsic mechanical properties, the nature and architecture of the ECM are also known to be important cues determining complex angiogenic and homeostatic processes, as reviewed elsewhere [283,284]. Similar to other cell types, mechanobiology studies have shown that adhesion and migration of ECs are also greatly impacted by substrate stiffness. In culture, cell adhesion is increased under physiological stresses, favouring anchoring and decreasing cell migration, while pathological stresses weaken this adhesion [50,285] (figure 3*b*). Moreover, when embedded and cultured in three-dimensional gels, ECs appear to migrate over longer distances in degradable matrices [286] while they spread inside soft ones [278], contrary to what is observed on flat two-dimensional substrates. This may have consequences for the specific migration of tip cells, as these transition from a curved two-dimensional support towards a three-dimensional matrix in which they elongate and form the sprout.

Interestingly, the mechanical properties of the ECM are modified during and by the process of angiogenesis. Dynamic changes have indeed been observed in the overall stiffness of tissues where neovessels actively form [287]. Angiogenic sprouting was linked to a local softening of the tissue associated with an increased MMP mRNA expression, while neovessel elongation was associated with a subsequent stiffening, explained by a decrease in proteolytic activity that was accompanied by an increase in the expression of genes related to ECM components and cell–ECM interaction.

Similar to the tensile stresses, substrate stiffness also greatly affects endothelial permeability, at least in part through cytoskeletal remodelling and exacerbation of inflammatory processes [50]. In this case, compliant matrices (4–10 kPa) are essential for the preservation of barrier function, while stiffer materials lead to an increased permeability due to disrupted adherens junctions and numerous intercellular gaps [288] (figure 3*b*), as well as increased formation of stress fibres [288,289]. Furthermore, age-related intimal stiffening has also been shown to increase EC permeability by upregulating cell contractility and modifying cell–cell junctions [290].

Until recently, the transition between maintenance and regression of new vessels was thought to depend exclusively on exposure to growth factors [264]. These particular chemical signals are now known to also be regulated by mechanical stimuli emanating from the matrix [263]. For example, matrix stiffness is known to generally enhance EC proliferation [274]. Matrix stiffening, in contrast, was found to promote a tumour vasculature phenotype, with more permeable and more tortuous vessels than healthy tissues [291]. Elucidating the mechanisms underlying vascular cell phenotype regulation by mechanical stresses requires understanding the intricate interactions between ECs and their matrices.

### 4.3. Cell matrix-generated forces

The mechanical coupling between ECs and their matrix is reciprocal: ECs sense and respond to mechanical cues, such as the tensile stresses and matrix properties described above, but they also generate stresses on the matrix. These cell-generated forces can alter the matrix, for instance through a strain-stiffening mechanism [292]. Interestingly, these forces also enable cells to communicate at long distances, up to dozens of micrometres, through matrix deformations [65,292] that appear to depend on the fibrous nature of the ECM [293]. This was recently demonstrated by studying mechanical interactions between cells at the matrix level using traction force microscopy (figure 3*c*). Califano & Reinhard-King [282] demonstrated that individual ECs exerted forces that propagated from the cell edges to the surrounding matrix, creating strains in the substrate. Recently, the Baker group showed that dynamic interactions between stalk cells and the neighbouring ECM were at the core of sprouting angiogenesis. Applying combined forces and proteolysis, sprout stalk cells indeed compact and degrade the ECM, opening a space for three-dimensional expansion that depends on the matrix density and the forces at play [294]. This matrix-mediated cell–cell mechanical communication was found to be critical to direct cell migration and organize the vascular network, guaranteeing viable function [65,292]. In particular, Ouyang *et al.* showed that ECs exploit the strain-stiffening and strain-aligning nature of a fibrous substrate to mechanically communicate at long distances and direct migration patterns by pulling on the ECM network [65] (figure 3*d*).

Since the discovery of the influence of mechanics on EC behaviour, investigating the mechanotransduction pathways involved in angiogenesis has been an intense field of study; a recent review summarizes all the mechanisms known to date [295]. Regarding the role of solid mechanics in angiogenesis, arguably one of the first identified mechanisms can be found in the work by Mammoto *et al.*, in 2009, where the existence of an ECM stiffness optimum was suggested for VEGF receptor (VEGFR2) expression in ECs [296]. Later, while searching for possible tissue engineering applications, it was proposed that the development of capillaries both *in vitro* and *in vivo* was not guided by the stiffness of the surrounding ECM *per se*, but that the matrix density was the global parameter that explains the mechanical influence of the ECM on angiogenesis, the orientation of its sprouts and their stability. In [297], denser collagen/fibronectin matrices indeed promoted the preferential orientation of the initial sprouts occurring during the destabilization stage in a direction parallel to the growth factor gradient, while less-dense materials presented more random or misaligned sprouts. In a follow-up study [298], insisting on the fact that the ECM density is related but not limited to stiffness, they developed an integrative method combining experiments and modelling to show that stable and more elongated sprouts are favoured by intermediate collagen densities of 1.2–1.9 mg ml^−1^. This range of concentration was identified as the one that strikes a balance between EC proliferation and migration. Low ECM densities, however, only permitted fragile sprouts, and dense matrices suppressed sprouting completely by impeding migration due to a high fibrillar entanglement. This influence of ECM density on angiogenesis was confirmed by a later study from the Hoying–Weiss group, who even developed a computational model that describes and predicts how three-dimensional neovessel topology is guided by ECM density [299]. A collagen density of 2 mg ml^−1^, similar to the previously cited range, allowed longer, more interconnected vessels with higher branching points and less free ends per unit length than higher densities. These studies, and others not cited here but mentioned in most of the cited papers, clearly indicate an active role of the interaction of neovessels with the stroma in angiogenesis, and this interaction is bidirectional. The growing vessels contract the ECM by exerting forces on the stromal fibrils, compact them into bundles and align them, while in turn, depending on its density, the ECM guides and orients the vessels, influencing their persistence length, branching and stability.

To more closely simulate *in vivo* conditions, a suspension of intact, isolated microvessel segments was cultured inside an ECM-derived matrix. This type of microvascular construct helped identify with more precision the exact three-dimensional mechanical interactions between cells and their ECM in the process of angiogenesis. Indeed, following culture in type-I collagen gels, the constructs can be implanted and, thanks to blood perfusion, can form hierarchical microcapillary networks that gradually adapt and remodel into a functional network, while offering a great tool for study [300]. Hoying, Weiss and their collaborators have successfully employed this implant technique in a series of biomedical applications and, using bioprinting techniques, imposed axial tissue deformations during the post-implantation remodelling phase; they showed that maintaining this external patterning stimulus allowed the imposition of a specific alignment of the final microvessel structure regardless of the initial architecture [301]. The removal of this constraint during the neovessel maturation stage resulted in a randomly oriented network. The alignment axes of the ECM fibrils thus guide cell shape and orientation and mediate the peak velocities of the sprouts (figure 3*a*). In sprouting angiogenesis, ECs invade a matrix of a certain density and mechanical stiffness, and this stromal environment is in turn modified by the neovessels. The understanding of how these bidirectional stresses influence the dynamics and shape of newly formed vessels is progressing, and better models can be constructed using microfluidic systems to elucidate or mimic this process in two dimensions and three dimensions, integrating fluid shear stress and soluble signals [276,302,303]. Several mathematical models based on experimental results have been developed and account for these complex mechanical interactions to describe changes in migration patterns during vessel formation [304,305]. Matrix deformations around angiogenic sprouts can also be measured experimentally using traction force microscopy, thereby providing a more precise understanding of neovessel patterning in both space and time. These measurements confirm a very dynamic pulling activity during sprout elongation [302–306]. Recently, a three-dimensional out-of-plane pulling activity of tip cells was identified *in vitro* [276] and was thought to be correlated with the fanlike reorientation of collagen fibrils near the tips of early-stage sprouts [307]. In [308], it was shown that the dynamic forces generated by the actomyosin machinery of ECs in three-dimensional fibrin gels were capable of bundling the stromal fibrils, hence increasing the ECM density locally, in a short period of only a few minutes. They also demonstrated that these differences in ECM ligand density could alter cell signalling and phenotype. Concerning the possible regionalization of cell phenotype, a model of tension and proliferation around tip cells [309] suggested that the tension exerted by the stalk cells created voids that could trigger the cell proliferation necessary for sprout elongation. In addition to this physical outcome, tissue deformation during angiogenesis may also be linked to the formation of a gradient of pro-angiogenic microenvironments, as shown in [310]. This regionalized heterogeneity in cellular density causes local differences in VEGF-A and VEGFR-2 expression and in cell proliferation rates. Finally, regarding the importance of the temporal impact of cell–ECM interactions, a recent study already cited above [246] has shown that in the context of tissue healing, the initiation time, magnitude and mode of compression of the ECM are all critical parameters that influence angiogenesis mechanotransduction. While immediate high-strain loading (of 30%) impeded angiogenesis by inhibiting early sprout tip cell selection genes, a delayed stress favoured neovessel formation, with a greater network length and a higher number of branches.

## 5. Challenges and perspectives

### 5.1. Coupled stimuli

Among the many difficulties associated with the *in vivo* study of angiogenesis, the coupled nature of different factors is of particular relevance to the biomechanical approach reviewed here. It is tempting to think of such a problem in linear terms (i.e. the effect of a combination of stimuli resulting in the sum of their individual effects); however, the integration of mechanical stimuli by cells has been shown to be a complex phenomenon [12,13,311–314]. Recent *in vitro* and *in vivo* studies point to active roles of different combined factors in the regulation of endothelial function and, more specifically, in angiogenesis. For instance, responses to shear stress can be influenced by both surface topography [315] and pressure [110]. These interactions can even lead to positive feedback loops that may prevent endothelial homeostasis [316]. Moreover, the mechanical response of ECs is bidirectional: EC response is dictated by the surrounding environment, which in turn is impacted by EC behaviour [65,292,294], adding another level of coupling.

Although most *in vitro* systems to date have focused on individual stimuli, we believe that the understanding of coupled effects is currently within our grasp thanks to the development of smart microfluidic systems that can be complemented with computational modelling [317–319]. In the context of *in vitro* platforms, we would like to highlight the work of Akbari *et al*. on the competing effects of IF, TF and LF [106]. Other examples include the work of Shirure *et al.*, which suggests that IF sensitivity is regulated by matrix stiffness [188] as well as the work of Abe *et al.* on the balance between VEGF concentration and IF (although coupled to TF) [169]. The interplay among matrix stiffness, shear stress and traction forces by cells was also studied to explore the possible existence of optimal mechanical conditions for new vessel formation and maintenance [302]. Finally, a highly illustrative example of the complex mechanical coupling that can exist in the microvasculature is provided by recent *in vitro* work involving the development of a microvessel-on-chip [320]. In this study, it was shown that a fraction of the LF crosses the endothelium and seeps into the viscoelastic and highly permeable collagen hydrogel that constitutes the microvessel wall. As a result, both the luminal pressure and the wall shear stress within the microvessel vary axially. The axial pressure variations, in turn, translate into changes in the microvessel diameter as a result of the deformation of the soft collagen hydrogel, which also has an effect on the pressure and wall shear stress fields. Finally, the TF associated with fluid seepage into the hydrogel changes pressure levels within the soft hydrogel which feeds back into the determination of the microvessel diameter. Deciphering the complex coupling described above is essential if such a microvessel system is to be used for understanding the role of mechanical factors in sprouting angiogenesis.

In addition to computational models that shed light on biochemical and mechanical stimuli [321,322], a particularly exciting direction is provided by emerging frameworks such as poroelasticity or active matter [46,323,324], which are opening new avenues of research in the coupling of stimuli during angiogenesis. We find active matter models especially promising, as they have already successfully been applied to other morphogenetic events such as epithelial cell extrusion [325] or hydra formation [326]. The active matter framework describes systems composed of individual active components that transform energy into mechanical work, an approach that appears to be particularly suited to mechanobiology. However, other possible approaches are also potentially promising. For instance, since mechanical properties of biological tissues have been shown to be associated with instabilities that determine morphogenesis [327–329], the notion that angiogenic sprouting can be viewed as an instability is certainly worth exploring. In this type of paradigm, different stimuli compete to either amplify or dampen the formation of sprouts [330], with stochasticity as a key player in the process [331]. Regarding sprout elongation, an analogy with jet stability that challenges the roles of tip and stalk cells has been proposed [332]. Based on this, and inspired by the theory of hydrodynamic instabilities [333], we see the need for the development of appropriate dimensionless numbers that describe the different regimes and potentially allow prediction of the angiogenic outcome under coupled mechanical and biochemical stimuli.

### 5.2. Biological scaling in angiogenesis: from organism to cell structures

Developing *in vitro* and *in silico* models that are truly pertinent to the angiogenic process is fraught with challenges. One of the difficulties stems from the multi-scale character of the factors affecting angiogenesis. Developing formulations that span the range from the subcellular scale to the scale of the entire organism constitutes a challenge that will undoubtedly require substantial effort from the research community.

To illustrate this need, we can start by considering the purpose of angiogenesis: *in vivo,* angiogenesis is vital for development, wound healing and tissue oxygenation [334]. Precise conditions need to be recapitulated *in vitro* in order to study the complex spatio-temporal mechanisms behind this process. While the majority of angiogenic stimuli are local, some, such as luminal shear stress or cyclic stretch resulting from the heartbeat, are systemic throughout the vascular network. Because of its critical role in meeting metabolic needs, the vascular system scales with the tissues it vascularizes [335]. A recent study demonstrated that blood vessels regulate epidermal proliferating clusters in skin by coordinating the stem cell population [336]. More generally, the branched architecture of blood vessels is thought to derive its existence from the need to optimize access from a single point to a surface or a volume [337]. However, how large-scale haemodynamic parameters impact angiogenesis at the cellular level remains unclear. In the particular case of a regenerative organ, such as the liver, mechanical stimuli are now known to mediate tissue growth: blood flow and pressure, in addition to biochemical signals, are extrinsic triggers of the regenerated organ and regulators of its size and vascularization [338].

Given the network nature of the vascular system and the constant evolution of its design space, it seems logical to think of its development in terms of topology optimization [339]. Moreover, its biological nature and the occurrence of vessel remodelling and pruning (i.e. its heuristic character) evoke the idea of using different types of optimization algorithms [340] to reproduce the vascular network. As can be inferred from the paragraph above, much work remains to unveil the constraints, loads and boundary conditions that arise from the spatio-temporal evolution of the angiogenic environment and that enable this type of approach. However, new hints are available concerning the relevant factors in this process. In light of recent findings, the roles of elastic energy dissipation and matrix viscoelasticity and viscoplasticity are critical in morphogenetic processes and in both individual and collective cellular behaviours [327,341,342]. It thus seems important to investigate these mechanisms in the context of angiogenesis [343]. Indeed, viscoelastic deformations of the ECM have been shown to play a major role in cell migration [344], vessel formation and stabilization [345,346].

Another concept that merits further attention is the scale at which mechanosensing and mechanotransduction act during angiogenesis. While some candidate mechanosensors such as the glycocalyx or mechanosensitive ion channels are essentially associated with individual cells, others such as cell–cell junctions are more suggestive of collective behaviour [36,347]. Recent evidence on the role of filopodia under VEGF stimulation [348] has set the course for a promising line of research. ECs sense and react to a VEGF stimulus individually, developing filopodia within seconds. Filopodia, in turn, increase EC sensitivity to VEGF, amplifying differences in the input signal. This mechanism determines cell fate, supported by Notch signalling in a subsequent stage [348]. Based on this study, we suggest that the effects of IF around a microvessel could be explained by a similar process, with filopodia acting as flow sensors.

## 6. Conclusion

Because of the role of the vasculature in the transport of oxygen, nutrients and metabolic products under both physiological and pathological conditions, it is fundamental to understand and control the formation of new blood vessels in biomedical and tissue engineering applications. While angiogenesis was initially thought to be driven exclusively by biochemical stimuli, most notably VEGF [349–352], research over the past two decades has established the central influence of mechanical factors. Both fluid and solid mechanical cues have been shown to greatly impact angiogenic sprouting through a variety of different mechanisms [156,168,263]. Here we reviewed the central role of ECs in those mechanisms specifically during the critical early phases of angiogenesis and highlighted outstanding questions, particularly in the context of EC responsiveness to multiple coupled stimuli. A key notion is that beyond individual response to mechanical cues, ECs also alter their environment, thereby providing cues to other cells or even to themselves [65,292]. A critical challenge will be to establish the role that other factors, including the presence of mural or parenchymal cells, play in the angiogenic process. Although *in vivo* experiments will continue to be essential for understanding the initiation and progression of angiogenesis, recent advances in physiologically relevant three-dimensional *in vitro* systems and in advanced computational models provide valuable tools in efforts aimed at understanding and controlling the angiogenic process.

## Data Availability

This article has no additional data.
